# *Croton hirtus L'Hér* Extract Prevents Inflammation in RAW264.7 Macrophages Via Inhibition of NF-κB Signaling Pathway

**DOI:** 10.4014/jmb.1908.08045

**Published:** 2019-11-18

**Authors:** Min Jeong Kim, Ju Gyeong Kim, Kong Many Sydara, Sang Woo Lee, Sung Keun Jung

**Affiliations:** 1 School of Food Science and Biotechnology, Kyungpook National University, Daegu 41566, Republic of Korea; 2 Ministry of Health, Institute of Traditional Medicine, Vientiane 116, Lao PDR; 3 International Biological Material Research Center, Korea Research Institute of Bioscience and Biotechnology, Daejeon 34141, Republic of Korea; 4 Institute of Agricultural Science & Technology, Kyungpook National University, Daegu 41566, Republic of Korea

**Keywords:** *Croton hirtus L'Hér*, inflammation, NF-κB, nutraceuticals, macrophage, nitric oxide

## Abstract

Consumption of anti-inflammatory nutraceuticals may help treat or prevent inflammation-related illnesses such as diabetes, cardiovascular disease, and cancer. This study evaluated the effect of *Croton hirtus L'Hér* extract (CHE) on lipopolysaccharide (LPS)-induced nitric oxide (NO) production and nuclear factor kappa-B (NF-κB) signaling cascades. CHE significantly suppressed LPS-induced NO production and inducible nitric oxide synthase (iNOS) expression in RAW264.7 macrophages, although cyclooxygenase (COX)-2 expression was not affected. CHE also suppressed LPS-induced IκB kinase (IKK), IκB, and p65 phosphorylation in RAW264.7 cells. Western blot and immunofluorescence assays of cytosol and nuclear p65 and the catalytic subunit of NF-κB showed that CHE suppressed LPS-induced p65 translocation from the cytosol to the nucleus. CHE also suppressed LPS-induced Interleukin (IL)-6 and tumor necrosis factor (TNF)-α production in RAW264.7 cells. These results suggest that CHE prevents NO-mediated inflammation by suppressing NF-κB and inflammatory cytokines.

## Introduction

Inflammation is an unbalanced defense response to infection or tissue injury [1]. Microorganisms can penetrate tissue injuries, and macrophages are essential to host immune defenses against these pathogens [2]. Toll-like receptor 4 (TLR4), a pattern recognition receptor (PRR), binds to bacterial lipopolysaccharide (LPS), the dominant structural element of the gram-negative bacteria outer membrane [3]. Detection of LPS through PRRs triggers a robust immune response, and excessive LPS can lead to septic shock and even death [4]. Nitric oxide (NO) is an important pro-inflammatory mediator regulated by inducible nitric oxide synthase (iNOS) [5]. Overproduction of NO leads to pronounced inflammation and tissue destruction, and the inhibition of NO signaling pathways is a promising strategy for attenuating inflammation [6].

Nuclear factor kappa-B (NF-κB) controls inflammatory gene expression, including genes for iNOS and cyclooxygenase (COX)-2 [7], and is the primary transcription factor implicated in LPS-mediated abnormal NO production in macrophages. NF-κB activation involves rapid and transient activation of IκB kinase (IKK) and IKK-mediated IκBα phosphorylation followed by IκBα degradation and translocation of NF-κB dimers, p50/p105 regulators, and the p65 catalytic subunit to the nucleus [8]. NF-κB-mediated, pro-inflammatory enzymes include iNOS, COX-2, and the inflammatory Interleukin (IL)-6 and tumor necrosis factor (TNF)-α [7]. Mitogen-activated protein kinases (MAPKs) also participate in the activation of NF-κB [9].

*Croton* is one of the largest genera of flowering plants, and it and other lianas are important secondary vegetation across a global range of ecologies [10]. Salatino *et al*. [11] reported that *Croton* metabolites contain anti-hypertensive, anti-cancer, anti-inflammatory, anti-microbial, and anti-viral properties. However, the anti-inflammatory and NF-κB signaling effects of *Croton hirtus L'Hér* extract have yet to be fully elucidated.

This study investigated the effects of CHE on LPS-induced NO production and NF-κB signaling pathways. Results indicated that CHE inhibits LPS-induced NO production and iNOS expression by suppressing NF-κB signaling pathways and inhibiting the expression of the inflammatory cytokines, including IL-6 and TNF-α.

## Materials and Methods

### Materials

Dulbecco’s Modified Eagle’s Medium (DMEM), fetal bovine serum (FBS), and antibiotics (penicillin/streptomycin solution) were obtained from Thermo Scientific HyClone (USA). LPS from *Escherichia coli* O111:B4 was purchased from Sigma-Aldrich (USA). Primary antibody against β-actin was purchased from Santa Cruz Biotech (USA). Antibodies against iNOS, COX-2, p-p65 (Ser536), p65, p-IκBα (Ser32), IκBα, p-IKKα/β (Ser176/180), IKKα, p-ERK1/2 (Thr202/Tyr204), ERK1/2, p-SAPK/JNK (Thr183/Tyr185), SAPK/JNK, p-p38 (Thr180/Tyr182), p38, and α/β-tubulin were purchased from Cell Signaling Technologies (USA). Anti-lamin B1 was obtained from Abcam (UK).

### CHE

*Croton hirtus L'Hér.* was collected in the Na Xay village, Xayboury district, Savannakhet province in Laos, having been identified there by Sangwoo Lee (Korea Research Institute of Bioscience and Biotechnology, Republic of Korea) in 2010. A voucher specimen (accession number KRIB 0033811) of the retained material is preserved at the herbarium of KRIBB. Dried and refined *Croton hirtus L'Hér* whole plant (71 g) was extracted with 1 L of 99.9% (v/v) methanol via repeated cycles of sonication (15 min) and rest (2 h) for 3 days at 45°C. The resultant product was filtered with non-fluorescent cotton and concentrated by rotary evaporator (N-1000SWD; EYELA, Japan) under reduced pressure at 45°C. After freeze drying, a total of 4.44 g of *Croton hirtus* methanol extract remained.

### Cell Culture

The murine RAW264.7 macrophage cell line was purchased from KCLRF (Korean Cell Line Research Foundation), the Korean Cell Line Bank (Republic of Korea) and maintained in DMEM containing 10% FBS and 1% antibiotics (100 U/ml penicillin and 100 µg/ml streptomycin) at 37°C in a 5% CO_2_ incubator (Thermo Scientific).

### Cell Viability

RAW264.7 cells at a concentration of 3 × 10^5^ cells/ml were seeded in 96-well plates and incubated at 37°C in a 5% CO_2_ incubator overnight. Cells were then treated with CHE at stepped concentrations for 24 h in 20 µl of 3-(4,5-dimethylthiazol-2-yl)-5-(3-carboxymethoxyphenyl)-2-(4-sulfophenyl)-2H-tetrazolium (MTS) reagent (Promega, USA) per well. After 1 h, absorbances were measured at 490 nm using a microplate reader (Bio-Rad Inc., USA).

### Nitrite Assay

Nitrite (NO_2_^-^) production was measured using colorimetric reactions with Griess reagents. RAW264.7 cells at a concentration of 3 × 10^5^ cells/ml were seeded in 96-well plates and incubated at 37°C for 24 h in a 5% CO_2_ incubator. Cells were pre-treated with CHE for 1 h followed by 1 µg/ml LPS for 24 h. Transferred nitrite accumulation in culture supernatants was evaluated with equal volumes of Griess reagent (0.2% N-(1-naphthyl)-ethylenediamine dihydrochloride (NED) and 1% sulfanilamide in 5% phosphoric acid). The reaction solution was incubated for 30 min, and then absorbances were measured at 550 nm using a microplate reader (Bio-Rad Inc.).

### Western Blot Analysis

RAW264.7 cells were seeded at a density of 1 × 10^6^ cells/ml on 60 mm cell culture dishes overnight. Cells were pretreated with CHE for 1 h before being stimulated with LPS (1 µg/ml) and incubated for stepped time periods. Cells were collected after incubation and washed twice with cold PBS. Total cell lysates were extracted with lysis buffer (Cell Signaling Technologies) mixed with a protease and phosphatase inhibitor cocktail (Thermo Scientific) and maintained on ice for 30 min with vortexing. Total protein content of the cell lysates was measured using a DC Protein Assay Kit reader (Bio-Rad Inc.) according to the manufacturer’s instructions. Harvested proteins were separated electrophoretically on a 10% sodium dodecyl sulfate–polyacrylamide gel (SDS-PAGE) and then transferred to a polyvinylidene difluoride (PVDF) membrane (Millipore, Immobilon-P transfer membrane, USA). The membrane was blocked in TBST buffer containing 5% skim milk for 1 h at room temperature. Then, specific primary antibodies were incubated with the membrane at 4°C overnight. After hybridization with horseradish peroxidase (HRP)-conjugated secondary antibody (Thermo Scientific) at room temperature for 1 h, protein bands were visualized using a chemiluminescence detection kit (ATTO, Japan) and GeneGnome XRQ NPC (Syngene, UK).

### Cytoplasmic and Nuclear Fractionation

RAW264.7 cells were seeded at a density of 1 × 10^6^ cells/ml on 100 mm cell culture dishes for 24 h in a 5% CO_2_ incubator. Cells were pre-treated with CHE for 1 h and then treated with 1 µg/ml LPS for 30 min. Cells were collected after incubation and washed twice with cold PBS. Separation of cytoplasmic and nuclear proteins was performed using NE-PER Nuclear and Cytoplasmic Extraction Reagents (Thermo Scientific) according to the manufacturer’s protocol. Separated proteins were then visualized with a western blot assay.

### Immunofluorescence

RAW264.7 cells were seeded at a density of 5 × 10^4^ cells/ml on 8-well chamber slides (ibidi, Germany). After incubation for 24 h, the medium was exchanged for medium containing CHE followed by another 1 h of incubation. Cells were then treated with LPS and incubated for 5 min. After incubation, cells were fixed with 4% formaldehyde, permeabilized with ice-cold MeOH, and treated with specific anti-p65 primary antibody overnight at 4°C. After washing, goat anti-rabbit IgG H&L conjugated to Alexa Fluor 488-conjugated labeled secondary antibodies (Abcam) was used for visualization. For nuclear staining, cells were stained with VECTASHIELD (Vector Laboratories, USA). The prepared cells were then observed under a confocal microscope (LSM700, Carl Zeiss, Germany), and images were recorded.

### Quantitative Reverse Transcription-PCR

Isolation of total RNA from the RAW264.7 cells was performed with RNAiso Plus (TAKARA, China) according to the manufacturer’s instructions. Synthesis of cDNA from isolated RNA was prepared with the ReverTra Ace qPCR RT Master Mix (Toyobo, Japan). Quantitative reverse transcription-PCR was performed using the SYBR Green Realtime PCR Master Mix (Toyobo) according to the manufacturer’s protocol. PCR primer sequences are listed in Table 1. Relative gene expressions were normalized to GAPDH via 2^−ΔΔCT^ method.

## Results

### Effect of CHE on LPS-Induced Nitrite Production and iNOS Expression in RAW264.7 Cells

**Fig. 1:**
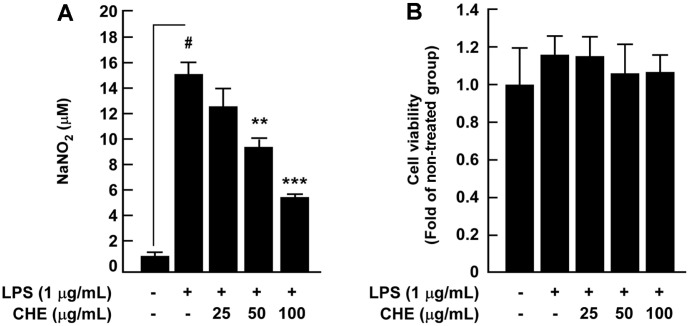
Effects of *Croton hirtus* L'Hér extract (CHE) on LPS-induced nitrite production and cell viability in RAW264.7 cells. (**A**) CHE suppressed LPS-induced nitrite production in RAW264.7 cells. Cells were pre-treated with CHE in the presence or absence of LPS (1 µg/ml) for 24 h. (**B**) CHE did not affect cell viability at the tested concentrations. The cells were treated with stepped concentrations of CHE for 24 h. Data are presented as mean ± SD of three independent experiments. ^#^*p* < 0.05 between control and LPS-exposed cells (no CHE); **p < 0.01 and ***p < 0.001.

**Fig. 2:**
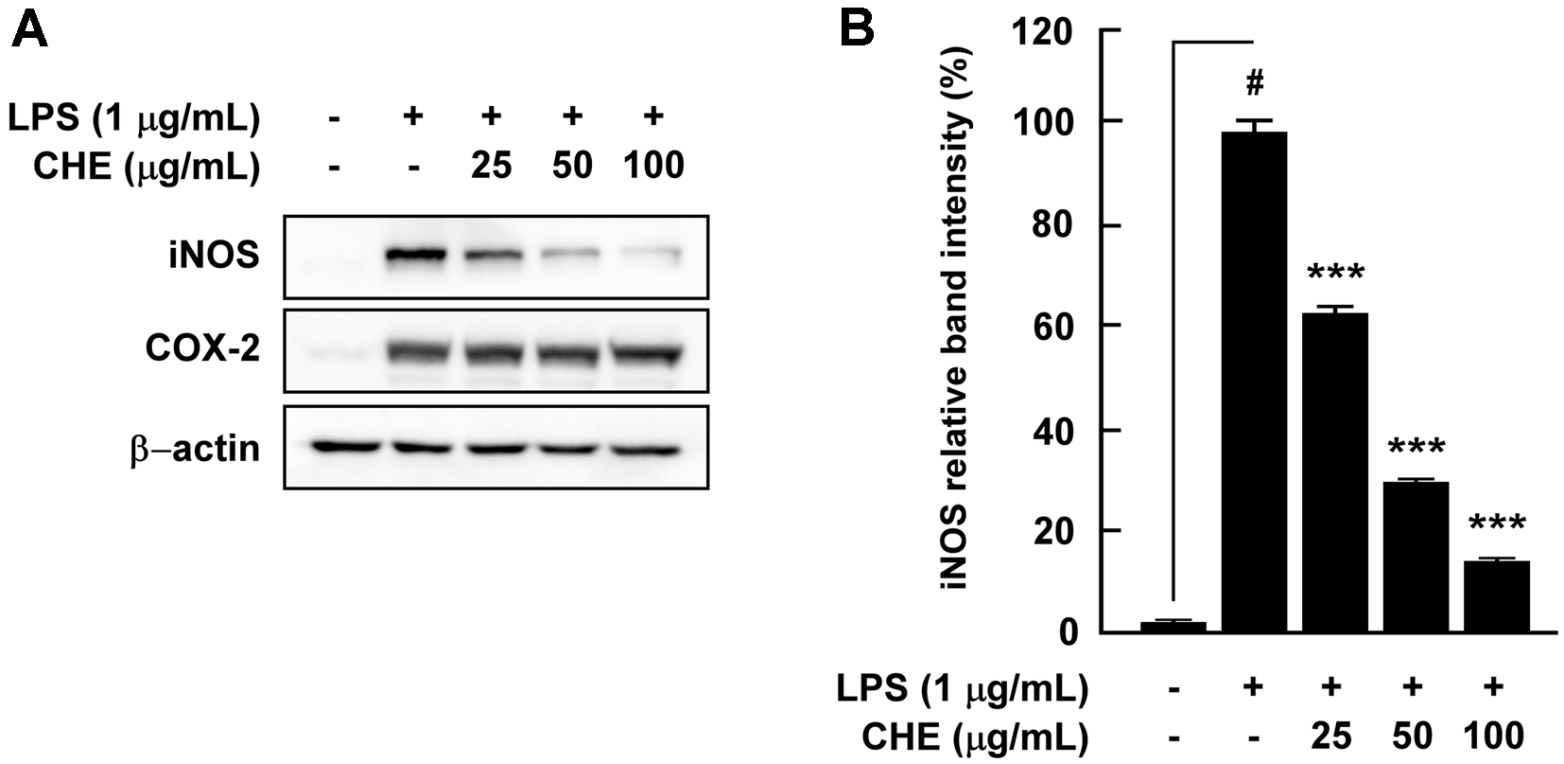
Effects of *Croton hirtus* L'Hér extract (CHE) on LPS-induced iNOS and COX-2 expression in RAW264.7 cells. (A) CHE suppressed LPS-induced iNOS but not COX-2 expression in RAW264.7 cells. (B) Quantification of iNOS suppression by CHE. Expression levels of iNOS, COX-2, and β-actin were determined by western blot. Data are presented as mean ± SD of three independent experiments. ^#^*p* < 0.05 between control and LPS-exposed cells (no CHE); ***p < 0.001.

Abnormal production of NO is critically involved in inflammation [6], and this study evaluated the effect of CHE on LPS-induced NO production in RAW264.7 cells. Results showed that CHE significantly inhibited LPS-induced nitrite accumulation in RAW264.7 cells in a dose-dependent manner (Fig. 1A). We evaluated the cytotoxic effects of CHE on RAW264.7 cells by treating them with stepped concentrations of CHE (0, 25, 50, and 100 µg/ml) for 24 h. Results demonstrated that CHE did not affect RAW264.7 cell viability at experimental concentrations (Fig. 1B). Since nitrite production was decreased by CHE, we then investigated the effect of CHE on LPS-induced iNOS and COX-2 expression, and western blot analysis results showed that CHE suppressed LPS-induced iNOS expression in a dose-dependent manner. COX-2 expression, however, was not altered (Fig. 2A).

### Effect of CHE on LPS-Induced NF-κB Signaling Pathways

**Fig. 3:**
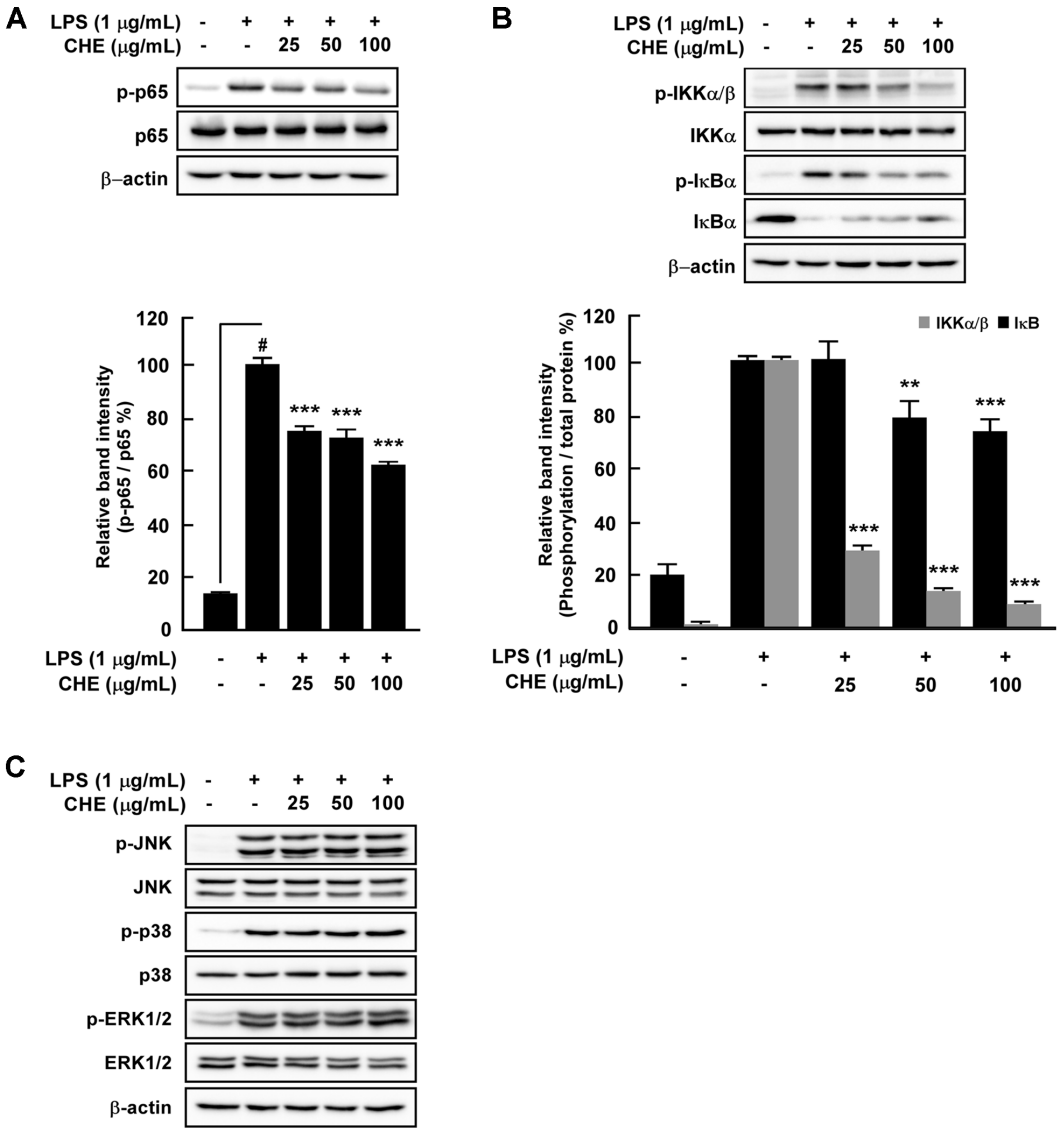
Effects of *Croton hirtus* L'Hér extract (CHE) on LPS-induced NF-κB and MAPK signaling pathways in RAW264.7 cells. (**A** and **B**) CHE inhibited LPS-induced phosphorylation of p65, IKK, and IκB in RAW264.7 cells. (**C**) CHE did not affect LPS-induced phosphorylation of p38, JNK 1/2, or ERK 1/2 in RAW264.7 cells. Cells were treated with the indicated concentrations of CHE and then stimulated with LPS (1 µg/ml). Levels of phosphorylation and expression were detected by western blot.

NF-κB regulates *inos* gene expression [12], and we evaluated whether CHE could affect LPS-induced NF-κB signaling pathways. Western blot analysis showed that CHE suppressed LPS-induced phosphorylation of IKK, IκB, and p65 in RAW264.7 cells (Figs. 3A and 3B). Activation of MAPKs is also important for LPS-induced NF-κB, AP-1 activation, and the subsequent activation of pro-inflammatory mediators such as iNOS and COX-2 in macrophages [13]. In this study, however, CHE did not affect the phosphorylation of p38, JNK, or ERK1/2 (Fig. 3C).

### Effect of CHE on LPS-Induced p65 Translocation from Cytosol to Nucleus in RAW264.7 Cells

**Fig. 4:**
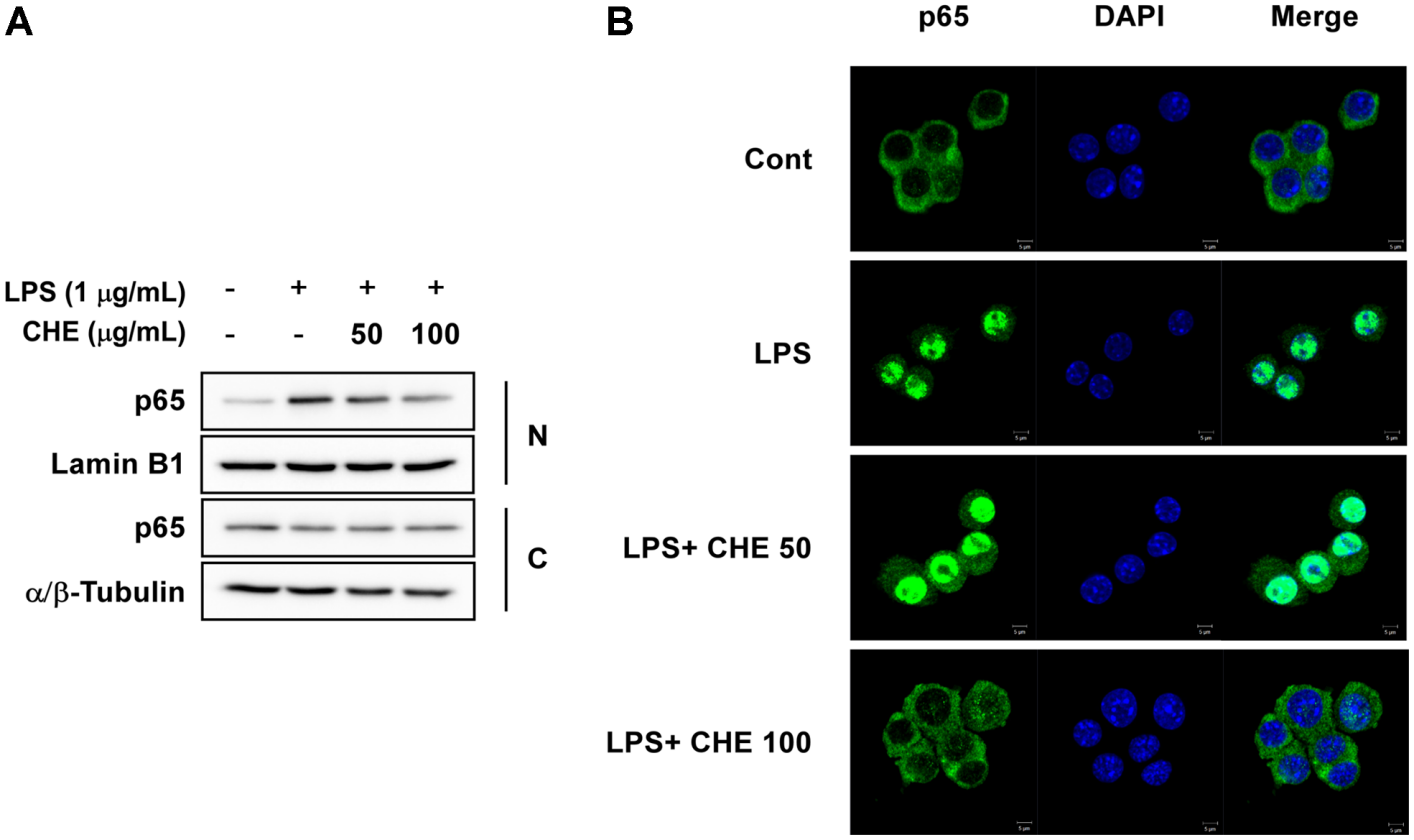
Effects of *Croton hirtus* L'Hér extract (CHE) on LPS-induced p65 NF-κB nuclear translocation in RAW264.7 cells. (**A**) CHE inhibited LPS-induced p65 expression in the nuclei of RAW264.7 cells. (**B**) Cells were treated with the indicated concentrations of CHE for 1 h and then stimulated with LPS (1 µg/ml) for 5 min. C, cytosol; N, nucleus

Translocation of the NF-κB p65 subunit from the cytosol to the nucleus is an indicator of NF-κB activation [14]. LPS stimulation resulted in increased p65 translocation to the nucleus over the control group, and CHE treatment appeared to suppress this LPS-induced p65 translocation from the cytosol (Fig. 4A). Immunofluorescence assays also demonstrated that CHE increased the localization of p65 to the cytosol compared to the LPS-only group (Fig. 4B).

### Effect of CHE on LPS-Induced IL-6 and TNF-α mRNA Expression in RAW264.7 Cells

**Fig. 5:**
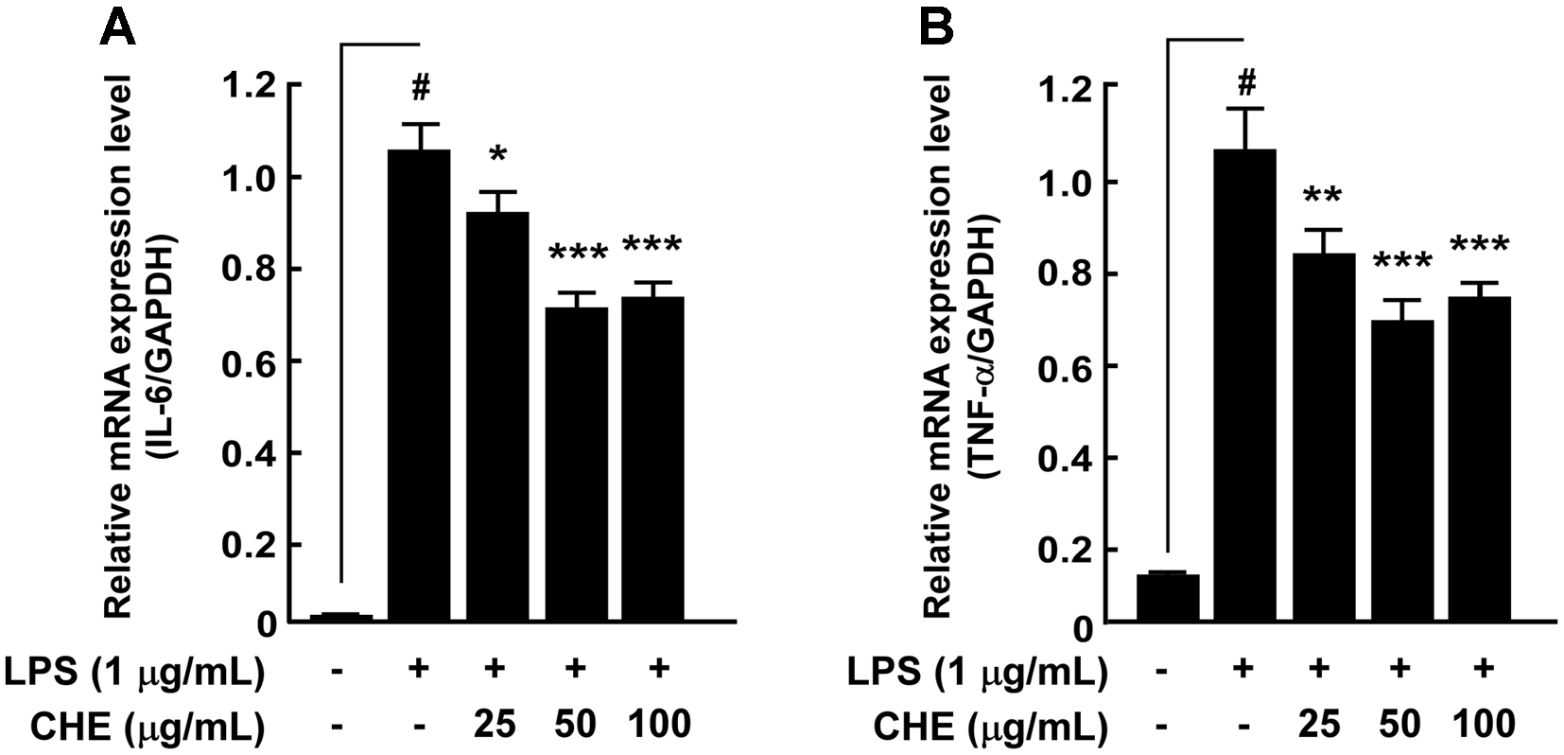
Effects of *Croton hirtus* L'Hér extract (CHE) on LPS-induced production of inflammatory cytokines in RAW264.7 cells. (**A**) CHE inhibited LPS-induced IL-6 and TNF-α mRNA expression in RAW264.7 cells. Cells were pretreated with CHE for 1 h and then stimulated with LPS for 24 h. Results were assessed by qRT-PCR. Data are presented as mean ± SD of three independent experiments. ^#^*p* < 0.05 between control and LPS-exposed cells (no CHE); *p < 0.05; **p < 0.01, and ***p < 0.001.

Activation of NF-κB results in the induction of genes encoding pro-inflammatory cytokines such as IL-6 and TNF-α [15]. We investigated the effect of CHE on LPS-induced IL-6 and TNF-α production in RAW264.7 cells with quantitative RT-PCR, and the results showed that CHE was significantly associated with the suppression of IL-6 and TNF-α mRNA expression in RAW264.7 cells in a dose-dependent manner (Fig. 5).

## Discussion

Alongside increased consumption of vegetables and fruits, nutraceuticals may help lower the risk of chronic disease through interaction with biological targets that cause inflammation [16, 17]. Chronic inflammation precedes diabetes, cardiovascular disease, and cancer, among many other diseases. Macrophage-mediated NO production is one of the most important factors involved in inflammation onset [18]. Although several studies [19, 20] have researched the capacity of known nutraceuticals to inhibit inflammation, the development of novel nutraceuticals with this facility remains important. We previously reported the possibility that agricultural and marine materials could be functional food materials with anti-inflammatory properties [21, 22]. Formerly, we used LPS treatment of RAW264.7 cells to screen 90 extracts for anti-inflammatory effects. CHE had the strongest inhibitory effect on LPS-induced NO production in RAW264.7 cells and was thus selected for further study (data not shown).

Abnormal expression of iNOS results in excessive production of NO, a critical inflammatory marker [23, 24]. Once the inhibitory effects of CHE on LPS-induced NO production were confirmed, we investigated the effect of CHE on LPS-induced iNOS expression and found that CHE suppressed LPS-induced iNOS expression in RAW264.7 cells. The expression of COX-2, which catalyzes the production of prostaglandins, has also been implicated in inflammation [25]; however, in this experiment, CHE had no effect on COX-2 expression. Similar effects have been reported for the herbal formula SC-E1 and extract of *Populus deltoides* [26, 27].

The NF-κB transcription factor regulates *inos* gene expression [28]. Under normal conditions, IκB regulates NF-κB activity via direct binding to NF-κB. Once LPS binds to TLR4, however, IKKα phosphorylates IκBα, stimulating an IκBα-induced proteasomal degradation process via ubiquitination [29]. Phosphorylation of p65, the catalytic subunit of NF-κB, at S276 facilitates NF-κB activity [30, 31]. As CHE was found to inhibit LPS-induced iNOS expression, we also evaluated the effect of CHE on the NF-κB signaling cascade. CHE suppressed LPS-induced phosphorylation of IKK and IκBα as well as p65 at S276 in RAW264.7 cells. After phosphorylation of IκBα by IKK, the NF-κB in the cytosol translocates to the nucleus and activates inflammatory genes, including those that code for iNOS, COX-2, and certain inflammatory cytokines [32]. We investigated the effect of CHE on LPS-induced p65 translocation with western blot followed by immunofluorescence assays of cytosolic and nuclear fractions, and the results showed that CHE inhibited LPS-induced p65 translocation to the nucleus in RAW264.7 cells. These combined findings suggest that the LPS-mediated IKK/IκBα/p65 signal cascade is blocked by CHE and that the accumulation of p65 in the cytosol results in the down-regulation of iNOS expression and subsequent NO production.

MAPKs also participate in inflammatory processes in macrophages [33]. MAPKs are composed of three types of kinases, extracellular-receptor kinases (ERK), the c-Jun N-terminal kinases/stress-activated protein kinases (JNK), and the p38 MAPKs. MAPK signaling can be activated by TLR4, which stimulates the nuclear translocation of NF-κB [34]. While MAPKs have been reported to participate in iNOS and NO production, in the present study, CHE did not affect the phosphorylation of MAPKs, suggesting that CHE targets other NO production pathways.

Cytokines are immunomodulators released in response to tissue injury or inflammation [35]. When an inflammatory cascade is induced, pro-inflammatory cytokines are upregulated by activated macrophages already involved in the inflammatory process [36]. When microbes containing LPS invade, macrophages respond by secreting inflammatory cytokines including IL-6 and TNF-α through the activation of NF-κB [36, 37]. Our results also confirmed that CHE inhibited IL-6 and TNF-α mRNA expression. Shim et al. [38] have shown that *Lysimachia clethroides* Duby has an inhibitory effect on LPS-induced NO and IL-6 production in macrophages and on IL-6 and TNF-α production in mouse bronchoalveolar lavage fluid. As CHE can inhibit LPS-induced inflammatory cytokines, we expect CHE to have a similar effect in the in vivo model, but further in vivo studies are needed to confirm our hypothesis.

Taken together, our results indicate that CHE inhibits LPS-induced NO production and iNOS expression, as well as production of the inflammatory cytokines IL-6 and TNF-α. This inhibition primarily occurs via suppression of the NF-κB signaling cascade and subsequent p65 translocation from the cytosol to the nucleus. This article is the first to report the preventive effects of CHE on NO-mediated inflammation in vitro.

## Figures and Tables

**Table 1:** Primer sequences.

Gene	Sense strand (5’-3’)	Antisense strand (3’-5’)
IL-6	TGG GAC TGA TGC TGG TGA CAA C	AGC CTC CGA CTT GTG AAG TGG T
TNF-α	TGG AAC TGG CAG AAG AGG CAC T	AGA GGC TGA GAC ATA GGC ACC G
GAPDH	ACT CCA CGA CAT ACT CAG C	TCA ACG GCA CAG TCA AGG
